# SLIMMER: a randomised controlled trial of diabetes prevention in Dutch primary health care: design and methods for process, effect, and economic evaluation

**DOI:** 10.1186/1471-2458-14-602

**Published:** 2014-06-14

**Authors:** Geerke Duijzer, Annemien Haveman-Nies, Sophia C Jansen, Josien ter Beek, Gerrit J Hiddink, Edith JM Feskens

**Affiliations:** 1 Division of Human Nutrition; Academic Collaborative Centre AGORA, Wageningen University, P.O. Box 8129, 6700 VE Wageningen, the Netherlands; 2 GGD Noord- en Oost-Gelderland (Community Health Service), P.O. Box 51, 7311 AB Apeldoorn, the Netherlands; 3 Strategic Communication, Sub-department Communication, Philosophy and Technology: Centre for Integrative Development, Social Sciences, Wageningen University, P.O. Box 8130, 6700 EW Wageningen, the Netherlands

**Keywords:** Type 2 diabetes mellitus, Prevention, Combined lifestyle intervention, Primary health care, Real-life setting, Evaluation design

## Abstract

**Background:**

Implementation of interventions in real-life settings requires a comprehensive evaluation approach. The aim of this article is to describe the evaluation design of the SLIMMER diabetes prevention intervention in a Dutch real-life setting.

**Methods/Design:**

The SLIMMER study is a randomised, controlled intervention study including subjects aged 40 through 70 years with impaired fasting glucose or high risk of diabetes. The 10-month SLIMMER intervention involves a dietary and physical activity intervention, including case management and a maintenance programme. The control group receives usual health care and written information about a healthy lifestyle. A logic model of change is composed to link intervention activities with intervention outcomes in a logical order. Primary outcome is fasting insulin. Measurements are performed at baseline and after 12 and 18 months and cover quality of life, cardio-metabolic risk factors (e.g. glucose tolerance, serum lipids, body fatness, and blood pressure), eating and physical activity behaviour, and behavioural determinants. A process evaluation gives insight in how the intervention was delivered and received by participants and health care professionals. The economic evaluation consists of a cost-effectiveness analysis and a cost-utility analysis. Costs are assessed from both a societal and health care perspective.

**Discussion:**

This study is expected to provide insight in the effectiveness, including its cost-effectiveness, and delivery of the SLIMMER diabetes prevention intervention conducted in Dutch primary health care. Results of this study provide valuable information for primary health care professionals, researchers, and policy makers.

**Trial registration:**

The SLIMMER study is registered with ClinicalTrials.gov (NCT02094911) since March 19, 2014.

## Background

Diabetes mellitus is one of the most challenging health problems of the 21st century [1]. Randomised controlled trials of lifestyle interventions have shown that a healthy diet and increased physical activity reduce the incidence of type 2 diabetes mellitus (T2DM) in impaired glucose tolerance patients [2-5]. This evidence calls for translation and implementation of diabetes prevention programmes in real-life settings to guide diabetes prevention policies. As real-life settings are complex and limited in finances and resources, it is a challenge to implement effective and sustainable interventions [6-8]. Multiple reviews that included studies conducted in several real-life settings, showed significant reductions in weight and waist circumference but inconclusive results for metabolic indicators of diabetes risk, such as blood glucose or HbA1c [7-10].

A comprehensive evaluation approach is required, as interventions in real-life settings are often complex and not delivered in tightly controlled environments [11,12]. Within this approach, the scope of evaluation research needs to broaden from assessing only effectiveness to also getting insight in the delivery of an intervention. This will provide insight in the so-called ‘black box’, that is identify aspects that explain what works, how, and why [11,12]. Therefore, studies need to include a process evaluation to establish the validity of the hypothesised causal processes for behaviour change and taxonomies can be used to describe behaviour change techniques used to modify these processes [13].

To date no effective diabetes prevention programme has been implemented in Dutch primary health care [14-17]. Therefore, the Study on Lifestyle intervention and Impaired glucose tolerance Maastricht (SLIM), revealing a 47% diabetes risk reduction [5], was translated into the SLIMMER intervention (SLIM iMplementation Experience Region Noord- en Oost-Gelderland). Translation of this intervention was done in a joint decision making process between SLIM intervention developers and local health care professionals [18]. Pilot-testing of the adapted intervention showed that implementation of the SLIMMER intervention was feasible in a Dutch real-life setting and that it was likely to achieve desired impact [19]. These results serve as input for the next step of broader implementation and evaluation of the intervention in a real-life setting.

The aim of this article is to describe the evaluation design of the SLIMMER diabetes prevention intervention in a Dutch real-life setting. This was done using a logic model describing the hypothesised causal pathway, including process indicators, behavioural determinants, and behavioural and health outcomes. The SLIMMER study will address the following research questions:

1. Which effects can be measured regarding behavioural determinants, eating and physical activity behaviour, health, and quality of life? (effect evaluation)

2. How is SLIMMER delivered and received in a real-life setting? (process evaluation)

3. How can results be interpreted in terms of costs and benefits? (economic evaluation)

## Methods/Design

### Logic model

**Figure 1:**
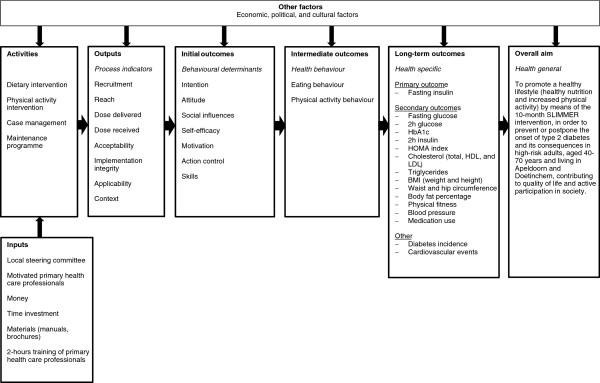
Logic model of change for the SLIMMER intervention.

For this study, a logic model of change is composed to link intervention activities, their mechanisms of change (i.e. behavioural determinants), expected behaviours, and intervention outcomes in a logical order. A logic model facilitates the understanding of intervention effectiveness and provides insights for further improvements [20,21]. Figure 1 shows the logic model of change for the SLIMMER intervention. The overall aim of the intervention is to prevent or postpone T2DM and its consequences and to increase quality of life. On the long-term, improvement in fasting insulin is taken as the primary outcome, whereas improvements in cardio-metabolic risk factors (e.g. glucose tolerance, serum lipids, body fatness, and blood pressure) are defined as secondary outcomes. Improvements in eating behaviour and physical activity behaviour are intermediate outcomes. Eating behaviour is measured as nutrient intake and food intake. Physical activity behaviour is operationalised as mode, frequency, duration, intensity, and activity score. Improvements in intention, attitude, social influences, self-efficacy, motivation, action control, and skills are formulated as initial outcomes. These outcomes are achieved if sufficient outputs are delivered in terms of recruitment, reach, dose delivered, dose received, acceptability, implementation integrity, applicability, and context.

### Study design

The SLIMMER study is a randomised, controlled intervention study, carried out in the Netherlands by a consortium of Wageningen University (WU, Wageningen) and the Community Health Service Noord- en Oost-Gelderland (GGD NOG, Apeldoorn). The total duration of the study is 1.5 years with an intervention period of 10 months. Recruitment of participants took place from October 2011 to September 2012. After baseline measurements, participants are randomly allocated to the intervention or control group, using block randomisation on the level of general practitioners (GPs) and stratification for gender. Couples are allocated to the same group to avoid contamination. Randomisation was performed by an independent dietician of the division of Human Nutrition (WU, Wageningen). The SLIMMER study has been registered with ClinicalTrials.gov (Identifier NCT02094911) since March 19, 2014. The WU Medical Ethics Committee approved the study protocol and all subjects gave their written informed consent before the start of the study.

### Setting

This study is carried out in Apeldoorn and Doetinchem, two average, middle-sized, Dutch cities, located in the eastern part of the Netherlands. The SLIMMER intervention is implemented in Dutch public health and primary health care, involving GPs and their practice nurses, dieticians, physiotherapists, and sports clubs. Within the study setting, GPs are organised in a formal network to deliver coordinated diabetes care. The majority of dieticians is employed by a home care organisation, only few are self-employed. No regional organisation or network for physiotherapists exists. All GPs have natural referral lines with at least one dietician and in most cases with one physiotherapy practice in the neighbourhood. This existing structure is used for implementation of the SLIMMER intervention. Furthermore, the project is coordinated by the community health service in close collaboration with both municipalities. Sports clubs are organised in a municipal sports stimulation organisation, which has an important role in the maintenance programme.

### Sample size calculation

The sample size calculation for this study is estimated based on changes in fasting insulin, observed in SLIM after one year [22]. In the SLIM study, mean difference in fasting insulin between groups was 2.9 mU/l with a standard deviation of 5.3 mU/l [22]. Because SLIMMER is conducted in a real-life setting instead of a controlled setting, it is estimated to achieve 75% of this result, that is an expected difference in fasting insulin between intervention and control group of 2.175 mU/l. Because we expect a larger SD in a real-life setting, we use 6 mU/l. To adjust for clusters (i.e. general practices), an intra-cluster correlation of 0.055 is used [23]. Based on results of SLIM [22] and the SLIMMER pilot study [19], we expect a drop-out rate of 10%. Assuming an alpha of 0.05, power of 80%, and two-sided test, a sample size of 145 subjects per group is required.

### Study population

GPs and practice nurses have selected patients aged 40 through 70 years suffering from impaired fasting glucose (IFG: i.e. fasting plasma glucose concentration 6.1-6.9 mmol/l [24]) in the past five years from their patient registration database. Patients are recruited using either laboratory glucose test or the Dutch Diabetes Risk Test [25]. Patients are considered for participation if they still suffer from IFG or if the test score indicates an elevated or high risk of T2DM (i.e. a score of ≥7 points). Inclusion and exclusion criteria (Table 1) are checked by GPs using electronic medical records. GPs have invited eligible patients to participate in the SLIMMER study. A short non-response survey is conducted in case patients are not willing to participate.

**Table 1:** Inclusion and exclusion criteria for the SLIMMER study

**Inclusion criteria**	Age 40 through 70 years
	Impaired fasting glucose (IFG; i.e. fasting plasma glucose concentration 6.1-6.9 mmol/l [24]) in the past 5 years according to the patient registration database, OR risk score ≥7 points based on the Dutch Diabetes Risk Test [25]
	Willing and able to participate in the study for at least 1.5 years
	Able to speak and understand the Dutch language
**Exclusion criteria**	Known diabetes mellitus
	Any chronic illness that makes 1.5-years survival improbable, interferes with glucose tolerance, or makes participation in a lifestyle intervention impossible
	Any severe cardiovascular disease (including history of cardiac dysrhythmia), unless general practitioner gives agreement
	Medication known to interfere with glucose tolerance (mainly systemic glucocorticoids and pituitary gland/hypothalamus hormones)
	Any mental or physical disability that will hinder participation in a lifestyle intervention
	Severe psychiatric disease
	Patients showing bad compliance in the past
	Participation in another regular vigorous exercise and/or dietary programme, i.e.:
	- Intensive physical activity programme: any physical activity programme offered by a physiotherapist and/or patients sporting at least three times a week at own initiative
	- Intensive dietary programme: patients who visited a dietician at least three times during the last year
	Patients who participated in the SLIMMER pilot study

### Lifestyle intervention programme

The SLIMMER intervention resembles the SLIM intervention [5], which was based on the Finnish Diabetes Prevention Study [4]. The SLIM intervention used a combination of theories, such as Stages of Change model [26] and Theory of Planned Behaviour [27], and tools, such as motivational interviewing [28] and goal setting. SLIMMER is a 10-month combined lifestyle intervention consisting of a dietary and physical activity component, including case management and a maintenance programme. The SLIMMER intervention conforms regular functioning and professional performance of Dutch GPs, practice nurses, dieticians, and physiotherapists. Minimal training and a detailed manual are provided during a two-hour SLIMMER kick-off training for health care professionals. In total, 25 general practices, 11 dieticians, nine physiotherapy practices, and 15 sports clubs are participating in the SLIMMER study. An overview of core tasks and competences of these professionals is given in Additional file 1. Details of the lifestyle intervention programme are described below.

### Dietary intervention

The dietary intervention is consisting of tailored dietary advice during individual consultations and one group session and is aimed to adopt, step by step, a sustainable healthy dietary pattern according to the Dutch dietary guidelines [29]. Furthermore, it is aimed to help participants to achieve 5-10% weight loss. Dietary recommendations are based on Dutch dietary guidelines [29], focussed on people at risk of developing diabetes. Dietary advice is given by a dietician from primary health care, trained in motivational interviewing [28] and using positive feedback. The number of consultations is flexible, ranging from five to eight (30–60 minutes per consultation; maximum of 4 hours per participant), and dependent on needs of participants. If desired, spouses could join consultations. In addition, the dietician organises one group session aimed at sharing experiences, motivating each other, and discussing the topic of label reading. Subjects are encouraged to drink less alcohol, quit smoking if necessary, increase daily physical activity, and to participate in the physical activity intervention. To stimulate self-management of participants, goals for behaviour change are set each consultation, evaluated in the next consultation, and if necessary adjusted. Halfway and at the end of the intervention, behaviour change is more extensively evaluated by dieticians to motivate participants, prevent drop-out, and discuss progression and goals.

### Physical activity intervention

The physical activity intervention is consisting of a combined aerobic and resistance exercise programme at the physiotherapist’s practice and is aimed to obtain and maintain an active lifestyle, that is moderate-intensity physical activity for at least 30 minutes per day on at least five days a week. Physical activity recommendations are based on Dutch guidelines for physical activity and type 2 diabetes [30]. Participants have free access to group-based training sessions and are stimulated to participate for at least one hour per week (maximum of two hours per week). Training sessions are given by a physiotherapist from primary health care and tailored to individual needs, desires, and opportunities. In addition, physiotherapists give tailored advice on how to increase physical activity in daily life (e.g. bicycling, walking) and goals are set. After three, six and ten months, behaviour change is monitored by physiotherapists (e.g. weight, waist circumference, and body fat percentage) aimed to motivate participants, prevent drop-out, and discuss progression and goals.

### Case management

Practice nurses are appointed as case managers of the intervention programme to enhance participant compliance and the feasibility of the implementation. They refer participants to the dietician and physiotherapist at the start of the intervention. Furthermore, they have the overview of the programme and work together with dieticians and physiotherapists. Four weeks after the start of the intervention and halfway the intervention, practice nurses contact dieticians, physiotherapists, and participants of the intervention group to facilitate contact among health care professionals, to detect and solve problems, and to motivate and support participants.

### Maintenance programme

A maintenance programme is added to the combined lifestyle intervention to guide participants in the process of maintaining lifestyle behaviour change in an independent and sustainable manner. This maintenance programme includes 1) intermediate evaluations (e.g. measurement of weight, waist circumference, and body fat percentage) by dieticians and physiotherapists to provide feedback and stimulate self-management; 2) sports clinics at local sports clubs to introduce participants to different sports activities; 3) final interviews with dieticians and physiotherapists at the end of the intervention to give positive feedback, discuss behaviour maintenance, and to set goals; 4) return visit with dieticians and physiotherapists to motivate and support participants in maintaining a healthy lifestyle; and 5) monitoring by practice nurses (i.e. discuss and monitor behaviour change during consultations at the general practice).

### Control group

Subjects in the control group receive usual health care as provided by GPs and practice nurses. Furthermore, they receive a minimal intervention at the start of the study, consisting of written information about beneficial effects of a healthy diet and increased physical activity, whereas no individual advice or programme is provided. No additional appointments are scheduled, apart from visits for follow-up measurements.

### Outcomes

Clinical assessments are performed by trained research assistants in research centres in Apeldoorn and Doetinchem. Furthermore, process and economic data are collected. Participants are measured at baseline (T0), after the intervention (12 months, T1), and six months after ending the intervention (18 months, T2). At each time point, participants are invited for two sessions on different days: one in the morning and one in the afternoon. Additional file 2 gives an overview of indicators, methods, and time points of the data collection.

### Effect evaluation

#### Socio-demographic characteristics

Participants fill in questionnaires on socio-demographic characteristics. Data on age, gender, education, ethnic background, marital status, job status, and smoking are collected according to standards of the national surveillance system for adults and the elderly in the Netherlands [31]. These national standards are based on best available scientific insights, experiences of local community health services, and expert opinions. Family history of diabetes is measured with a question from the Dutch Diabetes Risk Test [25]. Data on disease history are collected based on questions from the CoDAM study (Cohort study Diabetes and Atherosclerosis Maastricht) [32]. Non-response data (i.e. age, gender, reason for non-participation, perceived health, and education) are collected during the recruitment period by practice nurses.

#### Overall outcomes

Quality of life is assessed by the Short-Form Health Survey (SF-36), which proved to be a practical, reliable, and valid tool for both general and chronic disease populations in the Netherlands [33,34].

#### Long-term outcomes

A standard oral glucose tolerance test (OGTT; glucose load 75 g) is performed by a trained nurse after at least 10 hours of fasting. Fasting and 2 h plasma glucose levels, HbA1c, and serum lipids (cholesterol (total, HDL, LDL) and triglycerides) are determined at SHO laboratory in Velp, the Netherlands. For fasting and 2 h serum insulin, all blood samples are analysed within one run after 18 months. An index for insulin resistance is calculated from fasting plasma glucose and insulin concentration, using the homeostasis model assessment (HOMA index) [35]. Body mass index (BMI) is calculated as the ratio of weight and height squared (kg/m^2^). Waist circumference is obtained at the level midway between the lowest rib and the iliacal crest. Hip circumference is measured as the maximum circumference over the buttocks. Body fat percentage is measured by bio-impedance analysis (Tanita BC-418). Physical fitness is measured by the six-minute walk test [36], measuring the distance that participants walk within six minutes, which is an indicator of physical functional capacity. This is a simple, safe, and inexpensive sub-maximal exercise test [37]. In addition to distance, heart beat rate after six minutes and rating of perceived exertion are obtained using the 6–20 category Borg scale [38]. Blood pressure and heart beat rate at rest are measured using the Omron Digital Blood Pressure Monitor HEM-907. Self-reported use of medication (name, frequency, and duration of medication use) is determined using a questionnaire [39]. Diabetes incidence is based on data of self-reported medication use which are verified by GPs. Cardiovascular events are based on self-reported data measured by a questionnaire [32]. Procedures of measurements are described in protocols.

#### Intermediate outcomes

Eating behaviour is operationalised as nutrient intake and food intake. Nutrient intake is assessed by a validated Food Frequency Questionnaire (FFQ) [40,41]. FFQs are checked by trained research assistants. Average daily nutrient intakes are calculated by multiplying frequency of consumption by portion size and nutrient content per gram using the Dutch food composition table of 2006 [42]. Six food intake behaviours are formulated based on Dutch food-based dietary guidelines [43] and common dietician practices in the SLIMMER pilot study [19]: 1) eating 200 grams of fruit every day; 2) eating 200 grams of vegetables every day; 3) eating more whole grain bread; 4) eating less unhealthy snacks; 5) replacing fat bread spreads with lean bread spreads; and 6) drinking less soft drinks. These food intake behaviours are measured by an FFQ [40,41]. Physical activity behaviour is measured using the Short Questionnaire to Assess Health-enhancing physical activity (SQUASH), including questions on commuting activities, leisure time activities, household activities, and activities at work [44]. Physical activity behaviour is operationalised as mode, frequency, duration, intensity, and activity scores (i.e. total minutes of activity * intensity score). The SQUASH is a short, simple, reliable, and valid measure for categorising adults to their level of physical activity [44,45]. In addition, a question on sedentary behaviour is added, based on the Activity Questionnaire for Adults and Adolescents (AQuAA) [46].

#### Initial outcomes

A questionnaire is developed to measure behavioural determinants, as no validated questionnaires are available to measure determinants of specific nutrition and physical activity behaviours in adults at high risk of T2DM. To inform the development of the questionnaire, the Theoretical Domains Framework [47,48] is used in which behaviour change techniques, used in the SLIMMER intervention, are linked to behavioural determinants. The final questionnaire contains items on intention, attitude, social influences, self-efficacy, motivation, action control, and skills. Items are based on questions and scales described by Fishbein and Azjen [49], Lakerveld *et al.*[50], and Helmink *et al.*[51].

### Process evaluation

To assess how the SLIMMER intervention is delivered and received in a real-life setting, data from both participants and health care professionals are collected. A process evaluation plan is designed based on strategies of Steckler and Linnan [52], Saunders *et al.*[53], Nutbeam [54], and Wang *et al.*[55]. Process measures include recruitment, reach, dose delivered, dose received, acceptability, implementation integrity, applicability, and context. These process measures are assessed using the project logbook, non-response surveys, participant questionnaires, registration forms, attendance lists, and semi-structured interviews with health care professionals.

### Economic evaluation

Costs and effects of the SLIMMER intervention are compared with those of the usual care. Economic evaluation is performed from a societal perspective, taking all costs and benefits into account. In addition, a health care perspective is considered, in which only direct medical costs are taken into account. As in the effect evaluation, a time horizon of 1.5 years is used. Both a cost-effectiveness analysis (CEA) and a cost-utility analysis (CUA) are performed. The CEA presents clinical outcomes in terms of reduction of fasting insulin. The CUA presents outcomes in terms of quality-adjusted life years (QALYs), determined by the SF-6D health state classification, a preference-based single index derived from the SF-36 [56,57]. Intervention costs, health care costs, medication costs, patient costs, as well as productivity losses are assessed. In order to estimate intervention costs, time spent by different types of staff involved (practice nurses, dieticians, physiotherapists, providers of sports clinics, and project coordinator) and materials are identified by means of the project logbook, attendance lists, and registration forms. Volumes of health care use, medication use, absence from work, and other expenses are identified by means of participant questionnaires and registration forms. Costs associated with resources use are valued following Dutch guidelines for costing research within health economic evaluations [58,59]. If no standard cost prices are available, cost estimates from literature are used. All costs are expressed as year 2012 Euros. Where necessary, costs are indexed to the baseline year, as suggested in the Dutch manual [58,59]. Costs and effects in the second year are discounted at Dutch standard discounting rates of 4% (costs) and 1.5% (effects).

### Data analysis

Quantitative data analyses are performed following the intention-to-treat procedure. If necessary, data are transformed and analyses are adjusted for baseline measurements and possible differences between groups at baseline. To adjust for clustering on GP level, multilevel analyses are performed. To determine differences in effects between groups, multivariate analysis techniques are performed. Two-sided P values are calculated and a significance level of 0.05 is applied.

Qualitative data analyses are performed using an inductive approach [60]. Interviews with health care professionals are audiotaped and transcribed verbatim. All transcripts are read by two researchers individually to identify frequently emerging themes. These themes are used to create a coding scheme for analysis of data. Quotes are used to describe aspects of how the intervention is delivered and received.

Differences in costs and effects between intervention and control group are expressed as incremental cost-effectiveness ratios (ICERs). ICERs are plotted on a cost-effectiveness plane, a four quadrant diagram with a horizontal axis representing effect differences between the intervention and control group and the vertical axis representing costs differences between groups. In addition, a cost-effectiveness acceptability curve is constructed, which shows the probability that the SLIMMER intervention is cost-effective for a range of cost-effectiveness thresholds. Sensitivity analyses are conducted to assess robustness of results.

## Discussion

Implementation of diabetes prevention interventions in real-life settings requires a comprehensive evaluation approach. The design of the SLIMMER intervention described in this paper offers an appropriate evaluation strategy. Firstly, the logic model will facilitate understanding of the intervention effectiveness by assessing outcomes at several levels. Furthermore, the randomised design was adapted to be suitable for application in primary health care practice by incorporating block randomisation on GP level. Secondly, more attention is given to the process of intervention delivery, which is important for real-life, and thus less standardised, interventions. Thirdly, the economic evaluation will provide policy makers with valuable information on costs and benefits of an intervention.

In conclusion, this study is expected to provide insight in the effectiveness, including its cost-effectiveness, and delivery of the SLIMMER diabetes prevention intervention conducted in Dutch primary health care. Furthermore, it is expected that this study will facilitate our understanding of intervention components and characteristics that are associated with effectiveness. Results of this study provide valuable information for primary health care professionals, researchers, and policy makers.

## Abbreviations

AQuAA: Activity Questionnaire for Adults and Adolescents; BMI: Body mass index; CEA: Cost-effectiveness analysis; CODAM: Cohort on diabetes and atherosclerosis maastricht; CUA: Cost-utility analysis; FFQ: Food frequency questionnaire; GGD NOG: Gemeentelijke Gezondheidsdienst Noord- en Oost-Gelderland; GP: General practitioner; HbA1c: Glycated hemoglobin; HDL: High-density lipoprotein; ICER: Incremental Cost-effectiveness ratio; IFG: Impaired fasting glucose; LDL: Low-density lipoprotein; OGTT: Oral glucose tolerance test; QALY: Quality-adjusted life year; SF-36: Short-form health survey; SLIM: Study on lifestyle intervention and impaired glucose tolerance Maastricht; SLIMMER: SLIM iMplementation experience region Noord- en Oost-Gelderland; SQUASH: Short questionnaire to assess health-enhancing physical activity; T0: Baseline measurement; T1: Measurement after 12 months; T2: Measurement after 18 months; T2DM: Type 2 diabetes mellitus; WU: Wageningen University.

## Competing interests

The authors declare that they have no competing interests.

## Authors’ contributions

GD designed the evaluation study, collected and processed all data, and drafted the manuscript. SCJ and JTB participated in the study design and implementation in public health and primary health care, and helped to draft the manuscript. AH, GJH, and EJMF made major revisions to the manuscript. All authors contributed to the development of the SLIMMER intervention, and read and approved the final manuscript.

## Pre-publication history

The pre-publication history for this paper can be accessed here:

http://www.biomedcentral.com/1471-2458/14/602/prepub

## Supplementary Material

Additional file 1 Overview of core tasks and competences of primary health care professionals involved in the SLIMMER intervention. Click here for file

Additional file 2 Overview of indicators, methods, and time points of data collection. Click here for file

## References

[B1] International Diabetes Federation IDF Diabetes Atlas sixth edition 2013

[B2] Yoon U Kwok LL Magkidis A Efficacy of lifestyle interventions in reducing diabetes incidence in patients with impaired glucose tolerance: A systematic review of randomized controlled trials Metab Clin Exp 2013 62 2 303 314 10.1016/j.metabol.2012.07.009 22959500

[B3] Diabetes Prevention Program Research G 10-year follow-up of diabetes incidence and weight loss in the Diabetes Prevention Program Outcomes Study Lancet 2009 374 9702 1677 1686 19878986 10.1016/S0140-6736(09)61457-4 PMC3135022

[B4] Lindstrom J Ilanne-Parikka P Peltonen M Aunola S Eriksson JG Hemio K Hamalainen H Harkonen P Keinanen-Kiukaanniemi S Laakso M Louheranta A Mannelin M Paturi M Sundvall J Valle TT Uusitupa M Tuomilehto J Sustained reduction in the incidence of type 2 diabetes by lifestyle intervention: follow-up of the Finnish Diabetes Prevention Study Lancet 2006 368 9548 1673 1679 10.1016/S0140-6736(06)69701-8 17098085

[B5] Roumen C Corpeleijn E Feskens EJM Mensink M Saris WHM Blaak EE Impact of 3-year lifestyle intervention on postprandial glucose metabolism: The SLIM study Diabet Med 2008 25 5 597 605 10.1111/j.1464-5491.2008.02417.x 18445174

[B6] Garfield SA Malozowski S Chin MH Narayan KMV Glasgow RE Green LW Hiss RG Krumholz HM Considerations for diabetes translational research in real-world settings Diabetes Care 2003 26 9 2670 2674 10.2337/diacare.26.9.2670 12941736

[B7] Johnson M Jones R Freeman C Woods HB Gillett M Goyder E Payne N Can diabetes prevention programmes be translated effectively into real-world settings and still deliver improved outcomes? A synthesis of evidence Diabet Med 2013 30 1 3 15 10.1111/dme.12018 22998334 PMC3555428

[B8] Cardona-Morrell M Rychetnik L Morrell SL Espinel PT Bauman A Reduction of diabetes risk in routine clinical practice: Are physical activity and nutrition interventions feasible and are the outcomes from reference trials replicable? A systematic review and meta-analysis BMC Public Health 2010 10 653 10.1186/1471-2458-10-653 21029469 PMC2989959

[B9] Kahn R Davidson MB The reality of type 2 diabetes prevention Diabetes Care 2014 37 4 943 949 10.2337/dc13-1954 24652724 PMC3964495

[B10] Dunkley AJ Bodicoat DH Greaves CJ Russell C Yates T Davies MJ Khunti K Diabetes prevention in the real world: Effectiveness of pragmatic lifestyle interventions for the prevention of type 2 diabetes and of the impact of adherence to guideline recommendations - A systematic review and meta-analysis Diabetes Care 2014 37 4 922 933 10.2337/dc13-2195 24652723

[B11] O'Hara BJ Bauman AE Eakin EG King L Haas M Allman-Farinelli M Owen N Cardona-Morell M Farrell L Milat AJ Phongsavan P Evaluation Framework for Translational Research: Case Study of Australia’s Get Healthy Information and Coaching Service® Health Promot Pract 2013 14 3 380 389 10.1177/1524839912456024 22982704

[B12] Rychetnik L Frommer M Hawe P Shiell A Criteria for evaluating evidence on public health interventions J Epidemiol Community Health 2002 56 2 119 127 10.1136/jech.56.2.119 11812811 PMC1732065

[B13] Greaves CJ Sheppard KE Abraham C Hardeman W Roden M Evans PH Schwarz P Systematic review of reviews of intervention components associated with increased effectiveness in dietary and physical activity interventions BMC Public Health 2011 11 119 10.1186/1471-2458-11-119 21333011 PMC3048531

[B14] Vermunt PWA Milder IEJ Wielaard F De Vries JHM Baan CA Van Oers JAM Westert GP A lifestyle intervention to reduce Type 2 diabetes risk in Dutch primary care: 2.5-year results of a randomized controlled trial Diabet Med 2012 29 8 e223 e231 10.1111/j.1464-5491.2012.03648.x 22416789

[B15] Lakerveld J Bot SD Chinapaw MJ Van Tulder MW Kostense PJ Dekker JM Nijpels G Motivational interviewing and problem solving treatment to reduce type 2 diabetes and cardiovascular disease risk in real life: A randomized controlled trial Int J Behav Nutr Phys Act 2013 10 47 10.1186/1479-5868-10-47 23597082 PMC3639181

[B16] Jansen H Den Engelsen C Rutten GEHM Physical activity in patients with metabolic syndrome: At screening and three years thereafter Metab Syndr Relat Disord 2013 11 3 163 168 10.1089/met.2012.0110 23438154

[B17] Admiraal WM Vlaar EM Nierkens V Holleman F Middelkoop BJC Stronks K Van Valkengoed IGM Intensive Lifestyle Intervention in General Practice to Prevent Type 2 Diabetes among 18 to 60-Year-Old South Asians: 1-Year Effects on the Weight Status and Metabolic Profile of Participants in a Randomized Controlled Trial PLoS ONE 2013 8 7 10.1371/journal.pone.0068605 PMC3718785 23894322

[B18] Jansen SC Haveman-Nies A Duijzer G Ter Beek J Hiddink GJ Feskens EJ Adapting the SLIM diabetes prevention intervention to a Dutch real-life setting: Joint decision making by science and practice BMC Public Health 2013 13 1 10.1186/1471-2458-13-1 23656883 PMC3658924

[B19] Duijzer G Haveman-Nies A Jansen SC Ter Beek J Hiddink GJ Feskens EJM Feasibility and potential impact of the adapted SLIM diabetes prevention intervention in a Dutch real-life setting: the SLIMMER pilot study Patient Education and Counseling 2014 http://dx.doi.org/10.1016/j.pec.2014.05.024 10.1016/j.pec.2014.05.024 24993840

[B20] Bartholomew LK Mullen PD Five roles for using theory and evidence in the design and testing of behavior change interventions J Publ Health Dent 2011 71 SUPPL. 1 S20 S33 10.1111/j.1752-7325.2011.00223.x 21656946

[B21] Michie S Johnston M Francis J Hardeman W Eccles M From Theory to Intervention: Mapping Theoretically Derived Behavioural Determinants to Behaviour Change Techniques Appl Psychol 2008 57 4 660 680 10.1111/j.1464-0597.2008.00341.x

[B22] Mensink M Feskens EJM Saris WHM De Bruin TWA Blaak EE Study on lifestyle intervention and impaired glucose tolerance Maastricht (SLIM): Preliminary results after one year Int J Obes 2003 27 3 377 384 10.1038/sj.ijo.0802249 12629566

[B23] Littenberg B MacLean CD Intra-cluster correlation coefficients in adults with diabetes in primary care practices: The Vermont Diabetes Information System field survey BMC Med Res Methodol 2006 6 20 10.1186/1471-2288-6-20 16672056 PMC1513389

[B24] World Health Organization Definition and diagnosis of diabetes mellitus and intermediate hyperglycemia: report of a WHO/IDF consultation 2006 Geneva: World Health Organization

[B25] De Weerdt I Kuipers B Kok G ‘Kijk op diabetes’ met perspectief voor de toekomst 2007 Eindverslag van de eerste fase. In. Amersfoort: Nederlandse Diabetes Federatie

[B26] Prochaska JO Velicer WF The transtheoretical model of health behavior change AM J HEALTH PROMOT 1997 12 1 38 48 10.4278/0890-1171-12.1.38 10170434

[B27] Ajzen I The theory of planned behavior Organ Behav Hum Decis Process 1991 50 2 179 211 10.1016/0749-5978(91)90020-T

[B28] Miller WR Rollnick S Motivational interviewing: preparing people to change addictive behaviour 1991 New York: Guilford Press

[B29] Health Council of the Netherlands Guidelines for a healthy diet 2006. publication no. 2006/21E The Hague 2006

[B30] Praet SFE Van Uden C Hartgens F Savelberg HHCM Toereppel K De Bie RA KNGF-standaard Beweeginterventie diabetes mellitus type 2 [Royal Dutch Society for Physical Therapy’s guidelines on physical activity intervention type 2 diabetes mellitus] Amersfoort: Koninklijk Nederlands Genootschap voor Fysiotherapie 2009

[B31] Lokale en nationale monitor gezondheid - Indicatoren voor de monitor volksgezondheid [Local and national monitor health - Indicators for the monitor public health] https://www.monitorgezondheid.nl/volksindicatoren.aspx

[B32] Du H Van Der ADL Van Bakel MME Van Der Kallen CJH Blaak EE Van Greevenbroek MMJ Jansen EHJM Nijpels G Stehouwer CDA Dekker JM Feskens EJM Glycemic index and glycemic load in relation to food and nutrient intake and metabolic risk factors in a Dutch population Am J Clin Nutr 2008 87 3 655 661 18326604 10.1093/ajcn/87.3.655

[B33] Aaronson NK Muller M Cohen PDA Essink-Bot ML Fekkes M Sanderman R Sprangers MAG Te Velde A Verrips E Translation, validation, and norming of the Dutch language version of the SF-36 Health Survey in community and chronic disease populations J Clin Epidemiol 1998 51 11 1055 1068 10.1016/S0895-4356(98)00097-3 9817123

[B34] Ware JE Jr Sherbourne CD The MOS 36-item short-form health survey (SF-36). I. Conceptual framework and item selection Med Care 1992 30 6 473 483 10.1097/00005650-199206000-00002 1593914

[B35] Matthews DR Hosker JP Rudenski AS Naylor BA Treacher DF Turner RC Homeostasis model assessment: insulin resistance and β-cell function from fasting plasma glucose and insulin concentrations in man Diabetologia 1985 28 7 412 419 10.1007/BF00280883 3899825

[B36] Crapo RO Casaburi R Coates AL Enright PL MacIntyre NR McKay RT Johnson D Wanger JS Zeballos RJ Bittner V Mottram C ATS statement: Guidelines for the six-minute walk test Am J Respir Crit Care Med 2002 166 1 111 117 12091180 10.1164/ajrccm.166.1.at1102

[B37] Du H Newton PJ Salamonson Y Carrieri-Kohlman VL Davidson PM A review of the six-minute walk test: Its implication as a self-administered assessment tool Eur J Cardiovasc Nurs 2009 8 1 2 8 10.1016/j.ejcnurse.2008.07.001 18694656

[B38] Borg GAV Psychophysical bases of perceived exertion MED SCI SPORTS EXERC 1982 14 5 377 381 7154893

[B39] De Groot LCPGM Van Staveren WA Nutrition and the elderly A European collaborative study in cooperation with the World Health Organization Special Programme for Research on Aging (WHO-SPRA) and the International Union of Nutritional Sciences (IUNS), Committee on Geriatric Nutrition. Manual of Operations, Euronut Report 11 1988 Wageningen, the Netherlands: EURONUT

[B40] Streppel MT De Vries JH Meijboom S Beekman M De Craen AJ Slagboom PE Feskens EJ Relative validity of the food frequency questionnaire used to assess dietary intake in the Leiden Longevity Study Nutr J 2013 12 1 10.1186/1475-2891-12-1 23758629 PMC3680188

[B41] Siebelink E Geelen A De Vries JHM Self-reported energy intake by FFQ compared with actual energy intake to maintain body weight in 516 adults Br J Nutr 2011 106 2 274 281 10.1017/S0007114511000067 21338536

[B42] Stichting NEVO Dutch food composition table 2006 The Hague: Netherlands Nutrition Centre 2006

[B43] Food-based dietary guidelines [Richtlijnen Voedselkeuze] http://www.voedingscentrum.nl/Assets/Uploads/Documents/Voedingsvoorlichters/Richtlijnen_voedselkeuze_2011.pdf

[B44] Wendel-Vos GCW Schuit AJ Saris WHM Kromhout D Reproducibility and relative validity of the short questionnaire to assess health-enhancing physical activity J Clin Epidemiol 2003 56 12 1163 1169 10.1016/S0895-4356(03)00220-8 14680666

[B45] De Hollander EL Zwart L De Vries SI Wendel-Vos W The SQUASH was a more valid tool than the OBiN for categorizing adults according to the Dutch physical activity and the combined guideline J Clin Epidemiol 2012 65 1 73 81 10.1016/j.jclinepi.2011.05.005 21840174

[B46] Chinapaw MJM Slootmaker SM Schuit AJ Van Zuidam M Van Mechelen W Reliability and validity of the activity questionnaire for adults and adolescents (AQuAA) BMC Med Res Methodol 2009 9 1 10.1186/1471-2288-9-1 19664254 PMC3224705

[B47] Cane J O’Connor D Michie S Validation of the theoretical domains framework for use in behaviour change and implementation research Implement Sci 2012 7 1 10.1186/1748-5908-7-1 PMC3483008 22530986

[B48] Michie S Johnston M Abraham C Lawton R Parker D Walker A Making psychological theory useful for implementing evidence based practice: A consensus approach Qual Saf Health Care 2005 14 1 26 33 10.1136/qshc.2004.011155 15692000 PMC1743963

[B49] Fishbein M Ajzen I Predicting and changing behavior: The reasoned action approach 2010 New York: Psychology Press

[B50] Lakerveld J Bot SDM Chinapaw MJM Knol DL De Vet HCW Nijpels G Measuring pathways towards a healthier lifestyle in the Hoorn Prevention Study: The Determinants of Lifestyle Behavior Questionnaire (DLBQ) Patient Educ Couns 2011 85 2 e53 e58 10.1016/j.pec.2011.01.014 21296535

[B51] Helmink JHM Van Boekel LC Kremers SJP Pilot Beweegkuur overweight and obesity. Results of a follow-up measurement of participants Pilot BeweegKuur overgewicht & obesitas. Resultaten van een follow-up meting onder deelnemers 2010 Maastricht: Universiteit Maastricht

[B52] Steckler A Linnan L Process evaluation for public health interventions and research 2002 San Francisco: Jossey-Bass

[B53] Saunders RP Evans MH Joshi P Developing a process-evaluation plan for assessing health promotion program implementation: a how-to guide Health Promot Pract 2005 6 2 134 147 10.1177/1524839904273387 15855283

[B54] Nutbeam D Evaluating health promotion - progress, problems and solutions Health Promot Int 1998 13 1 27 44 10.1093/heapro/13.1.27

[B55] Wang S Moss JR Hiller JE Applicability and transferability of interventions in evidence-based public health Health Promot Int 2006 21 1 76 83 Epub 2005 Oct 2025 16249192 10.1093/heapro/dai025

[B56] Brazier J Roberts J Deverill M The estimation of a preference-based measure of health from the SF-36 J Health Econ 2002 21 2 271 292 10.1016/S0167-6296(01)00130-8 11939242

[B57] Brazier J Usherwood T Harper R Thomas K Deriving a preference-based single index from the UK SF-36 Health Survey J Clin Epidemiol 1998 51 11 1115 1128 10.1016/S0895-4356(98)00103-6 9817129

[B58] Hakkaart-van Roijen L Tan SS Bouwmans CAM Handleiding voor kostenonderzoek. Methoden en standaard kostprijzen voor economische evaluaties in de gezondheidszorg. Geactualiseerde versie 2010 Diemen: College voor Zorgverzekeringen 2010

[B59] Tan SS Bouwmans CAM Rutten FFH Hakkaart-Van Roijen L Update of the dutch manual for costing in economic evaluations Int J Technol Assess Health Care 2012 28 2 152 158 10.1017/S0266462312000062 22559757

[B60] Thomas DR A general inductive approach for analyzing qualitative evaluation data Am J Eval 2006 27 2 237 246 10.1177/1098214005283748

