# Supplementary material for: SLIMMER: a randomised controlled trial of diabetes prevention in Dutch primary health care: design and methods for process, effect, and economic evaluation
Source: BMC Public Health. 2014 Jun 14;14:602. doi: 10.1186/1471-2458-14-602 (PMC4067380; doi:10.1186/1471-2458-14-602)
Supplement: Additional file 1 — Overview of core tasks and competences of primary health care professionals involved in the SLIMMER intervention. [file 1471-2458-14-602-S1.pdf]

ADDITIONAL FILE 1. Overview of core tasks and competences of primary health care professionals involved in the SLIMMER intervention

1. Practice nurse

Goal

To select eligible patients with a high risk of type 2 diabetes mellitus (T2DM) from the electronic patient registration database and motivate them to participate in the SLIMMER study. Practice nurses inform patients on the risk of developing diabetes and how to reduce this risk by a healthy lifestyle.

Core tasks

1. To select patients with a high risk of T2DM, based on inclusion and exclusion criteria.
2. To inform patients about risk factors of T2DM and the importance of a healthy lifestyle; to motivate patients to participate in the SLIMMER study; to advise patients, taking into account their environment, needs, and opportunities.
3. To act as case manager to enhance participant compliance and the feasibility of the implementation. Practice nurses contact dieticians, physiotherapists, and participants of the intervention group to facilitate contact among health care professionals, to detect and solve problems, and to motivate and support participants.

Competences

- Stimulating patients towards a healthy lifestyle
- Good conversation and motivational skills
- Good listening skills
- Good coaching skills
- Monitoring progress of participants
- Being able to empathise with specific situation of participants
- Good coordination skills regarding role of case manager

2. Dietician

Goal

To guide participants towards an improved dietary pattern, based on Dutch dietary guidelines and focussed on people at risk of developing diabetes. Aim is to adopt a sustainable healthy dietary pattern step by step.

Core tasks

1. To carry out intakes to gather relevant information (e.g. dietary pattern, experiences, needs, environment) and set goals together with participants.
2. To formulate dietary advices to discuss during the individual consultations. It is important to support and motivate participants, to make an inventory of barriers and facilitators, and to set goals together with participants.
3. To organise a group session, aimed at sharing experiences, motivating each other, and discussing the topic of label reading (practice with comparing products on fat and sugar content).

Competences

- Conducting anamnesis of current dietary pattern
- Formulating tailored dietary advice
- Being able to empathise with specific situation of participants
- Stimulating participants towards a healthy lifestyle
- Good conversation and motivational skills (motivational interviewing techniques)
- Recognising and analysing barriers for healthy nutrition behaviour
- Applying dietary guidelines adequately
- Giving information on relation between nutrition and glucose tolerance
- Collecting data on dietary advice, progress and condition of participants
- Monitoring dietary pattern and compliance to dietary advices
- Detecting and tackling problems with new dietary pattern
- Building confidence with participants
- Improving participants' self-consciousness by listening and asking the right questions

- Guiding participants in a step-by-step behaviour change
- Helping participants get insight into their motivation
- Teaching participants new skills in a creative manner
- Organising and executing group session

3. <u>Physiotherapist</u>

<u>Goal</u>

To guide participants towards an improved physical activity pattern, based on Dutch guidelines for physical activity and type 2 diabetes. Aim is to obtain and maintain an active lifestyle, that is moderate-intensity physical activity for at least 30 minutes per day on at least five days a week, and (re-)discover pleasure of being physically active.

<u>Core tasks</u>

1. To carry out an intake to get insight in physical activity pattern, wishes, knowledge, opportunities, and experiences of participants.
2. To conceive a tailored physical activity programme based on information of the intake, consisting of weekly, group-based training lessons with both aerobic and resistance exercise. Besides individual coaching during the lessons, attention will be given to the group and team wise activities.
3. To stimulate and advise on tailored physical activity in daily life, taking into account opportunities and barriers of participants.
4. To guide participants to local sports clubs to maintain lifestyle behaviour change in an independent and sustainable manner. During the physical activity intervention, attention will be given to the period after ending the intervention.

<u>Competences</u>

- Being aware of Dutch guidelines for physical activity and type 2 diabetes
- Conducting anamnesis of current physical activity pattern
- Advising and stimulation of participants towards a healthy physical activity pattern

- Giving information on relation between physical activity and glucose tolerance
- Stimulating participants to participate in the SLIMMER exercise intervention
- Stimulating and advising participants to additional physical activity in daily life
- Detecting and tackling problems regarding the exercise intervention
- Monitoring compliance to physical activity advices
