# Supplementary material for: SLIMMER: a randomised controlled trial of diabetes prevention in Dutch primary health care: design and methods for process, effect, and economic evaluation
Source: BMC Public Health. 2014 Jun 14;14:602. doi: 10.1186/1471-2458-14-602 (PMC4067380; doi:10.1186/1471-2458-14-602)
Supplement: Additional file 2 — Overview of indicators, methods, and time points of data collection. [file 1471-2458-14-602-S2.pdf]

ADDITIONAL FILE 2. Overview of indicators, methods, and time points of data collection

Outcomes	Indicators	Method	Time points		
			**Baseline (T0)**	**12 months (T1)**	**18 months (T2)**
Socio-demographics	Age, gender, education, ethnic background,	Participant questionnaire [31]	X		
	Non-response data (age, gender, education, perceived health, reason for non-participation,)	Non-response survey [31]	X		
	Marital status, job status, smoking	Participant questionnaire [31]	X	X	X
	Family history of diabetes	Participant questionnaire [25]	X	X	X
	Disease history	Participant questionnaire [32]	X	X	X
Overall	Quality of life	SF-36 questionnaire [33, 34]	X	X	X
Long-term	Fasting and 2h insulin, fasting and 2h glucose, HbA1c, HOMA index	Oral Glucose Tolerance Test (OGTT)	X	X	X
	Cholesterol (total, HDL, LDL), triglycerides	Oral Glucose Tolerance Test (OGTT)	X	X	X
	Body weight, height, Body mass index (BMI), waist and hip circumference	Anthropometry	X	X	X
	Body fat percentage	Bio-impedance analysis (Tanita BC-418)	X	X	X

Duijzer, G. *et al.* SLIMMER: A randomised controlled trial of diabetes prevention in Dutch primary health care: design and methods for process, effect, and economic evaluation

	Physical fitness	Six-minute walk test [36]; Borg scale [38]	X	X	X
	Blood pressure	Omron Digital Blood Pressure Monitor HEM-907	X	X	X
	Medication use	Participant questionnaire [39]	X	X	X
	Diabetes incidence	Participant questionnaire [39]	X	X	X
	Cardiovascular events	Participant questionnaire [32]	X	X	X
	Economic indicators<br>- health care use<br>- absence from work	Participant questionnaire	X	X	X
Intermediate	Nutrient intake	Food Frequency Questionnaire [40, 41]	X	X	X
	Food intake behaviours<br>- eating 200 grams of fruit every day<br>- eating 200 grams of vegetables every day<br>- eating more whole grain bread<br>- eating less unhealthy snacks<br>- replacing fat bread spreads with lean bread spreads<br>- drinking less soft drinks	Food Frequency Questionnaire [40, 41]	X	X	X

	Physical activity behaviour<br>- mode<br>- frequency<br>- duration<br>- intensity<br>- activity score<br>- compliance to physical activity guidelines	SQUASH [44]	X	X	X
	Sedentary behaviour	AQuAA [46]	X	X	X
	Social participation	Participant questionnaire		X	X
Initial	Behavioural determinants<br>- intention<br>- attitude<br>- social influences<br>- self-efficacy<br>- motivation<br>- action control<br>- kills	Participant questionnaire (items based on [49-51]	X	X	X
Outputs	Recruitment (procedures used to approach and attract	Project logbook	X		

	participants at individual or organisational levels)				
	Reach (proportion of intended target audience that participated in an intervention)	Participant questionnaire, non-response survey, attendance lists	X	X	
	Dose delivered (number of amount of intended units of each intervention or component delivered or provided by interventionists)	Registration forms	X	X	X
	Dose received (extent to which participants actively engage with, interact with, are receptive to, and use materials or recommended resources)	Participant questionnaire, registration forms, attendance lists		X	X
	Acceptability (extent to which participants and health care professionals are satisfied with the intervention)	Participant questionnaire, semi-structured interviews with professionals		X	X
	Implementation integrity (extent to which the intervention was implemented as planned)	Semi-structured interviews with professionals	X	X	
	Applicability (extent to which an intervention process could be implemented in the real-life setting)	Semi-structured interviews with professionals	X	X	
	Context (aspects of the larger physical, social, and political environment that either directly or indirectly affects intervention implementation)	Participant questionnaire, semi-structured interviews with professionals	X	X	X

Economic	Intervention costs<br>- personnel (practice nurses, dieticians, physiotherapists, providers of sports clinics, project coordinator)<br>- materials	Project logbook, registration forms, attendance lists	X	X	X
	Patient costs (e.g. sports expenditures, time costs)	Participants questionnaire, registration forms	X	X	X
	Willingness-to-pay	Participant questionnaire	X	X	X
